# Anti-Vivisection Methods

**Published:** 1902-07-05

**Authors:** 


### The Institutional Workshop.

### ANTI-VIVISECTION METHODS.

Those peculiar people who have chosen to associate themselves with the National Anti-vivisection Society and to set themselves in opposition to such great charitable movements as the Hospital Sunday Fund and King Edward's Hospital Fund—movements initiated and carried on purely and exclusively for the benefit of the sick poor of London—have lately been making themselves fairly conspicuous, a thing which, no doubt, has to such people certain consolations, even though the comments passed upon them by honest men may be the reverse of complimentary. We have lately seen the strange spectacle of men disgracing themselves by taking advantage of the occasion of Hospital Sunday to distribute among the ministers of the various churches circulars containing among other matters the following impudent statement—"that not only does a very large portion of the money contributed by you go to the indirect support of animal torture, as well as of the system of experimentation on human beings which is its logical and inevitable result, but that a small portion of the money which you venture to offer on the altars of that God of Love, before Whom not one sparrow is forgotten, is directly and avowedly employed in the torture of animals," a statement the absolute falsity of which is apparent to everyone who knows anything about the matter. All this seems to have been done for the sake of diverting the money of the charitable from the great purpose for which they were to be called together, into the coffers of an absurd proposal to establish what these silly people describe as an anti-vivisection hospital, as if it were possible in the treatment of disease to hold oneself aloof from knowledge gained by experiments on animals, or to refuse treatment to a child gasping at the point of death because, forsooth, a horse had been used in the production of the remedy or because this remedy had been tested on a rabbit before being used on a suffering child. But a still more barefaced attempt on the sympathies and purses of the foolish and soft-hearted has lately come to our notice in which Archdeacon Wilberforce, a dignitary of the Church, a man whose duty it is to teach honesty and probity to all men, actually took advantage of Hospital Sunday, and of a Hospital-Sunday congregation, drawn together for the purpose of supporting the great hospitals of London, to collect money for a little hospital in the New Kent Road run on the cheap and simple plan of giving its patients no meat. Not that this appears to be its chief virtue; Archdeacon Wilberforce's interest in the concern apparently lying in the fact that the Hon. Stephen Coleridge is its chairman, and in the pretence that it "is entirely free from any connection with vivisection." Of course any such claim is absurd. We should much like to hear from those members of the staff of this hospital who hold appointments at other institutions, how they manage to do their honest best for the patients at these different places if it be true that, while they use for the one set all the knowledge they have gathered from every source, they restrict themselves in treating the patients at the other to knowledge and methods which are "entirely free from any connection with vivisection." In any case it is somewhat amusing to find that Mr. Coleridge, who poses as an opponent of experiments on animals, should be the chairman of a vegetarian hospital which exists for the very purpose of trying a very doubtful experiment on human beings.

Mr. Coleridge himself furnishes another example of the notoriety which has fallen upon anti-vivisectionists and their methods during the last week or two, for Mr. Coleridge has been attempting to clear his character by bringing an action for libel against a man who had traduced him, and he has failed to get a verdict, which shows pretty plainly what common-sense people think of the rhodomontade in which anti-vivisectionists so constantly indulge and of the honesty of their propaganda. The facts of the case were simple enough. After a meeting of the National Anti-vivisection Society which had been held at Derby, the Rev. David Macdonald, a Nonconformist minister in that town, wrote a letter which was published in the local press, in which was the following paragraph:—

"Some letters in the *Morning Post* during this week may interest your readers as an example of Mr. Coleridge's method of controversy. They may also help the Bishop to understand why men, lovers of animals and lovers of truth, will have nothing to do with his society."

That was the first libel complained of, and for that Mr. Coleridge demanded an apology. In answer to this demand, however, he got a still stronger letter which was also published. It contained the following stinging passage:—

"At the meeting on the 23rd I heard the speakers for your society. It seemed to me that in your letters and in their speeches there was a use of the method technically known as 'suppression of the true and suggestion of the false.' I wrote to the *Derby Express* that people could see in your letters your 'method of controversy,' that the same method was used by the speakers. Then I named the method, and gave two examples of its use. And I assert that the use of the method is sufficient to make men who, like myself, claim to be lovers of animals and lovers of truth have nothing to do with your society."

In other words it described Mr. Coleridge as an untruthful person. Here it may be well to remark that Mr. Coleridge allowed his counsel, in opening his case and in speaking of vivisection, to make the astounding remark that "this society was not opposed to it so long as it was properly carried out." This was made without any protest or correction on the part of the plaintiff, and we must suppose that it was this that led the learned judge later on to say "What he appears to mean by the suppression of truth is that you call yourselves anti-vivisectionists, while you really are in favour of vivisection," a remark both witty and *à propos*, seeing that Mr. Coleridge's great complaint was of being called an untruthful person.

The strange thing about this trial was that after all that has been written about the anti-vivisection methods in papers of wide circulation and by people of well-known scientific position, Mr. Coleridge should have taken the trouble to attack a Nonconformist minister down at Derby. However, Mr. Macdonald proved to be of tougher fibre than the anti-vivisectionists expected, and even when promised that Mr. Coleridge would not ask for costs, he declined to withdraw his words. It is not every day that we can agree with Mr. Coleridge; we cannot but agree with him however in some of his evidence. He swore, for example, that in the first letter above referred to, Mr. Macdonald accused him of being a liar, and we cannot say that he did not. Again he swore that he considered the letters were "damaging to his character" and we cannot deny that they were. For all that the jury stopped the case, giving him neither damages nor verdict, and Mr. Justice Grantham added "I quite agree with you." Finally as a last hope of getting some consolation Mr. Coleridge's counsel asked whether the jury all agreed, but even here no comfort was to be had, for the answer was "Yes, we all agree."

